# Supplementary material for: Learning clinical networks from medical records based on information estimates in mixed-type data
Source: PLoS Comput Biol. 2020 May 18;16(5):e1007866. doi: 10.1371/journal.pcbi.1007866 (PMC7259796; doi:10.1371/journal.pcbi.1007866)
Supplement: S1 File — Benchmark data generation (continuous and discrete variables). Performance measures. Benchmark parameter tuning. Resource availability. (PDF) [file pcbi.1007866.s001.pdf]

SUPPORTING INFORMATION

for manuscript

Learning clinical networks from medical records based on information estimates in mixed-type data

Vincent Cabeli, Louis Verny, Nadir Sella, Guido Uguzzoni, Marc Verny, Hervé Isambert

Supplementary Materials and Methods

Benchmark data generation

In this section we describe the generation of datasets used for the mixed-type (Fig. 3, Figs. S11 and S12) and continuous (Figs. S14 and S15) benchmarks, and implemented in the R script provided as supplementary material. First, the underlying DAG models were randomly drawn from the space of all possible DAGs [1], allowing for a maximum degree of 4 neighbours. Datasets were generated following the causal order of the generated DAG using non-linear structural equations models (SEMs), as outlined below.

The first nodes in the causal order have no parents, their distributions are sampled either from Gaussian mixtures of 1 to 5 modes (with equal σ) for continuous nodes or with a uniform random sampling of 2 to 4 categorical levels. The distribution of every other node X was generated as a function of its parents $\mathrm{Pa}(X)$ plus some Gaussian noise as, $X = f(\mathrm{Pa}(X)) + \epsilon$. Depending on whether X and its parents are continuous or categorical, different models were used:

- **Continuous variable** X

  The causal relationship between a continuous node X and its continuous parents $\mathrm{Pa}_c(X)$ plus their pairwise interaction products $I(\mathrm{Pa}_c(X))$ was modeled using polynomials: $X = R(\sum_{Y_i \in \{\mathrm{Pa}_c(X) \cup I(\mathrm{Pa}_c(X))\}} R(Y_i, -1, 1)^{c_i} + \epsilon, 0, 1)$ with c_i chosen in $[1, 3]$, ϵ some Gaussian noise with variance depending on the number of parents and c_i, and $R(X, \min, \max)$ a re-scaling function so that the distribution X is in the range $[\min, \max]$. In the case of mixed-type parents, *i.e.* with some continuous and some discrete parent variables, sets of c_i were drawn for each combination of the discrete parents $\mathrm{Pa}_d(X)$. If all its parents are categorical, a child node is categorical as well. Finally, the distribution of a continuous node has an equal probability to be transformed with a non-linear function, e^X, $\sin(X)$ or $\cos(X)$, or to be retained as is.

- **Discrete variable** X

  The continuous parents of a discrete node are first discretized by attributing categorical levels to the distinct peaks if there are any (see Fig. S13), or using equal frequency binning with $\log(N)$ bins otherwise. The discrete distribution of the node X is then drawn from random sampling with probability w_i for the ith level of X, where each combination of the levels of $\mathrm{Pa}_d(X)$ are associated to a different set of probabilities $\{w_i\}$.

Performance measures

For the evaluation, the network reconstruction was treated as a binary classification task and classical performance measures, precision, recall and F-score, were used, based

on the numbers of true *versus* false positive (TP *vs* FP) edges and true *versus* false negative (TN *vs* FN) edges. The precision $Prec = TP/(TP + FP)$ indicates how reliable the edges of the reconstructed network are. This measure does not indicate, however, which fraction of the true edges are detected, which corresponds to the sensitivity or recall of the reconstruction, $Rec = TP/(TP + FN)$. Finally, the F-score is a global performance measure, which is defined as the harmonic mean of precision and recall measures: $Fscore = 2Prec \times Rec/(Prec + Rec)$. In particular, a Fscore of 1 implies a perfect reconstruction without FP nor FN edges.

In order to measure how well the orientations of the edges match those of the true DAG, we also define the orientation-dependent counts $TP' = TP - TP_{misorient}$ and $FP' = FP + TP_{misorient}$ with $TP_{misorient}$ corresponding to all true positive edges of the skeleton with different orientation/non-orientation status as in the true Complete Partially Directed Acyclic Graph (CPDAG). Here, CPDAG refers to the equivalence class of the true DAG, which is taken as the benchmark reference since different DAGs might be equivalent from the data point of view (*i.e.* if and only if they have the same skeleton and the same v-structures). The CPDAG precision, recall and F-score were then computed with the orientation-dependent TP' and FP'.

Benchmark parameter tuning

The performances of some methods rely on tunable parameters which typically determine the sparsity of the inferred graph. In contrast, miic uses a complexity term derived from the normalised maximum likelihood and is essentially parameter-free. Although in real world applications the best settings cannot be known for certain, meaningful comparisons can only be done after each method has been properly parameterized. Here we detail the steps taken to find the best parameters for each benchmark setting.

For the mixed-type benchmarks, ranges of parameters for both CausalMGM [2] and MXM [3] methods were tested, and their best results (*i.e.* best F-scores) obtained for a given sample size (N) and percentage of continuous node (p_c) were compared to miic results. For CausalMGM, the λ sparsity parameter for all edge types (discrete-discrete, continuous-continuous, discrete-continuous) was tested in $\{0.050, 0.073, 0.108, 0.158, 0.232, 0.341, 0.500\}$. For MXM, the significance threshold α used for the various independence tests was tested in $\{0.001, 0.005, 0.01, 0.05, 0.1, 0.2\}$.

For the continuous benchmarks, we first optimized each method on separate simulations to find a good approximating function for the best parameter $\hat{\alpha} = f_p(N)$. The best values for the α_N parameter of PC gaussian, PC rank, CAM for sample sizes N spaced evenly on a log scale between 100 and $10,000$ were first found using a zeroth order parameter optimization implemented in `dlib` [4, 5]. Then, the function f_p was fitted as a second order polynomial over all values of N and α_N. kPC (using the Hilbert-Schmidt independence criterion with gamma approximation [6, 7]) was not optimized so extensively, due to its much longer execution time, and was only tested for the conservative values of α: 0.05 and 0.15.

Resource availability

- **MIIC R package** for ***mixed-type data*** is available at this URL: https://miic.curie.fr/download/miic_mixed.tar.gz
- **MIIC online server** for ***mixed-type data*** is accessible here: https://miic.curie.fr/workbench_mixed.php

References

1. Melançon G, Philippe F. Generating connected acyclic digraphs uniformly at random. Information Processing Letters. 2004;90(4):209–213.

2. Sedgewick AJ, Buschur K, Shi I, Ramsey JD, Raghu VK, Manatakis DV, et al. Mixed graphical models for integrative causal analysis with application to chronic lung disease diagnosis and prognosis. Bioinformatics. 2018;doi:10.1093/bioinformatics/bty769.

3. Tsagris M, Borboudakis G, Lagani V, Tsamardinos I. Constraint-based causal discovery with mixed data. International Journal of Data Science and Analytics. 2018;6(1):19–30.

4. Malherbe C, Vayatis N. Global optimization of lipschitz functions. In: Proceedings of the 34th International Conference on Machine Learning-Volume 70. JMLR. org; 2017. p. 2314–2323.

5. King DE. Dlib-ml: A Machine Learning Toolkit. Journal of Machine Learning Research. 2009;10:1755–1758.

6. Gretton A, Herbrich R, Smola A, Bousquet O, Schölkopf B. Kernel methods for measuring independence. Journal of Machine Learning Research. 2005;6(Dec):2075–2129.

7. Gretton A, Spirtes P, Tillman RE. Nonlinear directed acyclic structure learning with weakly additive noise models. In: Advances in neural information processing systems; 2009. p. 1847–1855.
