# Supplementary material for: Learning clinical networks from medical records based on information estimates in mixed-type data
Source: PLoS Comput Biol. 2020 May 18;16(5):e1007866. doi: 10.1371/journal.pcbi.1007866 (PMC7259796; doi:10.1371/journal.pcbi.1007866)
Supplement: Attachment — Submitted filename: responses_to_reviewers.pdf [file pcbi.1007866.s018.pdf]

We would like to thank the reviewers for their constructive comments and suggestions. We address all of them in our responses below and in the revised manuscript (revisions in red).

Reviewer 1

The authors present a method for computing the mutual information between mixed variables by finding an optimal binning strategy. They demonstrate that the method is competitive with state-of-the-art methods for estimating mutual information between mixed variables and that it has a particular advantage as an independence test. They then apply this mutual information estimator to graphical model structure learning and demonstrate good performance on benchmark data as well as present a case study application to a medical data.

The method and application are technically sound and well-presented.

My two main concerns are:

1. In the author summary and introduction an impression is built up that no methods exist for computing mutual information for mixed variables. The authors are clearly aware of these methods (references 15-17), however, the mention of these methods is pushed down deep into the benchmarking subsection of the results section. These must be brought to the forefront (be referenced in the introduction) as not to misrepresent the state of the art.

   Following the reviewer's suggestion, we now mention these recent methods for computing mutual information for mixed variables in the Introduction as well as in the benchmarking subsection of the Results section.

2. There's no explanation of the principle by which "latent variables" are suggested in the graphical model, i.e. what makes an edge suggest mediation by a latent variable vs a simple correlation/anticorrelation edge. If this is a post-hoc decision in light of expert knowledge the text needs to be explicit about that.

   We have now added a paragraph on the presence of latent variables in MIIC inferred networks within the new Methods section (see below). Latent variables, while unobserved in the available dataset, manifest themselves in the form of bidirected edges in MIIC inferred networks. The rationale to infer such bidirected edges is not based on post-hoc decision in light of expert knowledge but is actually learnt from the available data as reported with methodological details in our 2017 PLoS Comput Biol paper (Verny *et al* 2017).

Reviewer 2

Review of the PLOS Computational Biology manuscript PCOMPBIOL-D-19-01535 ”Learning clinical networks from medical records based on information estimates in mixed-type data” by V Cabeli, L Verny, N Sella, G Uguzzoni, M Verny, H Isambert

Summary:

This paper presents an extension of the MIIC network learning algorithm for mixed-type (i.e. both continuous and categorical) data. This new approach relies on a new estimation procedure for the (conditional) Mutual Information (MI) for such mixed-type data, also introduced in this manuscript. After introducing the need and relevance of such methods especially in the context of medical records, the authors present new methodological developments for estimating (conditional) MI, that is suitable for mixed-type data, and illustrate its good performance on benchmark synthetic. Then the authors outline their extension of the MIIC algorithm for mixed data, briefly benchmark it, and present an extensive application to medical records of elderly patients with cognitive disorders. Finally, a short discussion quickly highlights the conclusions from that application.

General Comments:

This manuscript presents an interesting and timely new method for estimating network from mixed-type data such as medical records. While the manuscript is well written, the structure is a bit confusing and impedes both its readability and assessment: first it lacks a materials and methods section which should contains the methodological developments that are currently being presented alongside simulations benchmarks and application in the Results section; secondly the Discussion section should be broader and better acknowledge the assumptions and limitations made by the proposed method. Besides, I have questions concerning the guarantees offered by the proposed method and the assumptions required, as those are not clearly outlined in the manuscript. In particular, I wonder how the authors deal with the scaling of the MI and how it impacts edge pruning and filtering in their network inference. My questions to the authors are detailed below.

Major issues:

1. The MI is an unbounded positive quantity, therefore one of the difficulties of using MI for inferring networks from mixed-type data is the scaling of the MI that will usually varies depending on the variable type (binary, categorical, continuous...). This aspect should be discussed in the manuscript. In particular, the MI for categorical variables tends to increase with the number of categories. How do the proposed method deals

with this when i) pruning (and filtering) the edges of the inferred network ? ii) representing the association strength such as in Figure 4 ?

While the range of (conditional) mutual information indeed depends on the variable types, it is not a difficulty for our method. In fact, our approach exploits these quantitative differences in multivariate information to prioritize all its algorithmic decisions, based on Information Theory principles, while taking into account the finite size of the dataset.

In particular, the assessment of variable independence or dependency integrates the number of categories for discrete variables and the number of optimized partitions for continuous variables through a normalized maximum likelihood (NML) complexity cost.

Furthermore, as outlined in the Methods section of the revised manuscript (and detailed in Verny *et al* 2017), (conditional) mutual information estimates integrating NML complexity costs are related to i) the probability to remove the corresponding edges (which can be used to filter the initial skeleton) and represent ii) the association strength between variables (which is displayed through the width of individual edges in MIIC networks).

2. The manuscript lacks a method section. New methodological development should be in a specific Methods section, with a first subsections presenting the new approach for approximating partial MI in mixed-data and a second one presenting the extension of the MIIC algorithm.

   We now have a Methods section as requested by this reviewer.

3. Discussion section should discuss the whole scope of the manuscript, including assumptions and limitations of the proposed approach for learning network from mixed-data, as well as synthetic benchmark results and application.

   We now have a Discussion section covering the whole scope of the manuscript.

4. Page 4 line 82-33, the authors seem to make an assumption on the partitioning cut-points that should be clarified, especially if it is required for their approximation to be accurate.

   There is actually no particular assumption on the partitioning cut-points of continuous variables, just the recognition that the number of cut points needs to be specified and thus encoded in the model within the frame of the Minimum Description Length (MDL) principle, as first argued in Kontkanen *et al* JMLR 2007 paper on MDL-optimal histograms for continuous variables. Hence, in absence of specific priors for any partition with r bins, the model index should be encoded with a uniform distribution over all partitions with the same number of bins. As there are $\binom{N-1}{r-1}$ ways to choose $r-1$ out of $N-1$ possible cut points, it leads to a codelength of $\log\binom{N-1}{r-1}$ to specify the partition of a continuous variable into r bins,

which corresponds to the additional term in the complexity cost for each continuous variable Eq. 12 (previously Eq. 6).

5. The authors should detail a bit more how they derived equation 7 or provide a reference.

   We now provide more detailed insights into the dynamic programming scheme for mutual information optimisation and clarify the different terms of Eq. 13 (previously Eq. 7).

6. It is unclear whether there are guarantees for the convergence of the proposed optimization procedure presented at the bottom of page 4, or if this is more of a heuristic procedure that works in practice.

   As discussed in the revised manuscript, there is a guarantee for a convergence towards a local maximum of information, although not necessarily the global maximum (unless there is only a single continuous variable). In this sense, the general optimization scheme can indeed be seen as an heuristic procedure that works in practice.

7. The authors should describe what are X and Y represented on Figure 2 and how they are generated in the synthetic benchmark (this is somewhat explained in the SI but should be mentioned and clarified in the main manuscript).

   We now describe in more details the data of Figure 2 in the main text as well as in SI.

8. Page 6 the authors alludes to the capacity of their approach to identify (conditional) independence. Could they clarify how do they characterize independence from (part) MI in my experience this can be difficult in practice, even with resampling procedures?

   Independence or conditional independence is characterized by a negative or null (conditional) mutual information including finite size effects, *i.e.* $X \perp\!\!\!\perp Y \mid Z \iff I'(X;Y|Z) \leqslant 0$, as first introduced in Affeldt *et al.* 2015.

   For continuous or mixed-type variables, however, the optimization scheme typically returns $I'(X;Y|Z) = 0$ *exactly* for (conditional) independence, which corresponds to a single bin for X and/or Y.

9. I command the author in making a software available for their method in the form of the R package miic. However, I was unable to find (and so test) the mentioned discretizeMutual function neither from the CRAN version of the package or on GitHub. The authors should provide an url for the code of the proposed approach.

   As mentionned on page 2 of the SI, we provide on our website all source codes including the discretizeMutual function (`https://miic.curie.fr/download/miic_mixed.tar.gz`). We will update both the CRAN and github versions of the R package MIIC as soon as we can include the reference to the present paper.

Minor issues:

1. Page 2 line 36, cause-effet should appear in English
2. Figure 1 & 3 should have a linetype/color legend and should be readable in black & white
3. Page 6 line 142 KSG acroym is never defined

Thank you for pointing out these minor issues to us. We have fixed them in the revised manuscript.
