# Supplementary material for: Learning clinical networks from medical records based on information estimates in mixed-type data
Source: PLoS Comput Biol. 2020 May 18;16(5):e1007866. doi: 10.1371/journal.pcbi.1007866 (PMC7259796; doi:10.1371/journal.pcbi.1007866)
Supplement: Attachment — Submitted filename: responses_to_reviewers2.pdf [file pcbi.1007866.s019.pdf]

Reconstruction, Analysis and Evolution of Biological Networks
Institut Curie, CNRS UMR168, PSL Research University
26, rue d'Ulm, 75005 Paris, France
Tel: +33 1 56 24 64 74 herve.isambert@curie.fr

PLoS Computational Biology
Editorial Office

April 7, 2020

Dear Editor,

Please find the second revised version of our manuscript "**Learning clinical networks from medical records based on information estimates in mixed-type data**" submitted to *PLoS Computational Biology* with changes in red in the main text.

This lattest version includes as initially planned and requested by reviewer 2: *i)* the benchmark scripts used to generate Figures 2 & 3 (which are provided at a github repository) and *ii)* the fully deidentified clinical datasets analyzed in Figure 4 (which is provided as a supplementary dataset table with our manuscript). We append below our replies to the reviewers' comments on first revision of our manuscript.

In addition, it is also our intention to provide all the other scripts used to generate the benchmark results presented in Supplementary Figures, however, due to the strict covid-19 lockdown currently in place in Paris and at our Institute, we have not been able to retrieve these scripts, which are stored in one of our desktop computers without remote access. We would like to reassure you that these few remaining scripts will be uploaded to the same github repository as the main figure scripts, as soon as we regain access to them.

On behalf of the authors, I would like to thank you again for your interest in this manucript and hope that you will formally accept it for publication in *PLoS Computational Biology* at your earliest convenience.

Yours sincerely,

Dr Hervé Isambert,
Group leader, Institut Curie
Directeur de recherche, CNRS

Reviewer 1

1. This is a review of the revised version of PCOMPBIOL-D-19-01535R1 'Learning clinical networks from medical records based on information estimates in mixed-type data'

   The authors present a method for computing the mutual information between mixed variables by finding an optimal binning strategy. They demonstrate that the method is competitive with state-of-the-art methods for estimating mutual information between mixed variables and that it has a particular advantage as an independence test. They then apply this mutual information estimator to graphical model structure learning and demonstrate good performance on benchmark data as well as present a case study application to a medical data.

   The method and application are technically sound and well-presented.

   The authors' revisions have fully addressed all the concerns I had in my prior review.

   I recommend publication as dissemination of this method will be of value to the research community.

   We thank the reviewer for his time and expertise in reviewing our manuscript.

Reviewer 2

1. The authors have adequaltely adressed all my comments. I recommend that the authors pay extra attention to provide **all data underlying the figures and results presented in the manuscript** in their final submission (especially regarding Fig 2 and 4).

   We thank the reviewer for his/her time and expertise in reviewing our manuscript. This lattest version includes as initially planned and requested by this reviewer: *i)* the benchmark scripts used to generate Figures 2 & 3 (which are provided at a github repository) and *ii)* the fully deidentified clinical datasets analyzed in Figure 4 (which is provided as a supplementary dataset table with our manuscript).
